# From Exams to Engagement: Evaluating Project-Based Learning in Introductory Biostatistics With R for Public Health Students

**DOI:** 10.1177/23821205251376539

**Published:** 2025-09-24

**Authors:** Kevin C Lutz, Sean G Young, Lindsey Chambers, L Joseph Su

**Affiliations:** 1 Peter O’Donnell Jr School of Public Health, The University of Texas Southwestern Medical Center, Dallas, TX, USA

**Keywords:** assessments, biostatistics education, programming in R, project-based learning, public health education

## Abstract

**Objective:**

To evaluate the impact of replacing a traditional midterm exam with a collaborative project-based assessment on student performance and engagement in an introductory graduate-level biostatistics course for public health students using R programming.

**Methods:**

We conducted a retrospective study comparing 2 semesters of the same course at a US school of public health. In Fall 2023, students completed traditional midterm and final exams. In Fall 2024, the midterm exam was replaced with a structured project-based assessment, while the final exam format remained unchanged. Student performance was compared using midterm scores, final exam scores, overall course grades, and course evaluations. Additionally, we used the results of a postproject survey that students in the project-based cohort had completed to assess engagement, confidence, and satisfaction.

**Results:**

Students in the project-based cohort (Fall 2024) had significantly higher and more consistent midterm scores (median 94.0 vs 91.1; *P* = *.*01) and final exam scores (median 93.7 vs 86.0; *P* = *.*03) than the exam-based cohort (Fall 2023). Final course grades were also higher and more consistent in the project- based cohort. Survey responses indicated high student satisfaction, improved confidence in data analysis and R programming, and increased appreciation for real-world applications. Students also identified challenges related to group dynamics and time management.

**Conclusion:**

Replacing a traditional exam with a collaborative project-based assessment in biostatistics significantly improved student performance, engagement, and satisfaction. These findings support project-based learning as a valuable pedagogical strategy in public health education, particularly for developing applied data analysis skills with R.

## Introduction

In public health, statistical literacy is crucial for tasks such as analyzing epidemiological data, evaluating interventions, and informing policy decisions. Formal training in public health is lacking among many government public health workers, with estimates suggesting only 14% hold such qualifications,^1^ which implies that there could be gaps in essential competencies like biostatistics and data analysis. The increasing complexity of health data requires professionals who can accurately interpret statistical information. Statistics education today faces a range of challenges that hinder student understanding and engagement such as struggling to grasp abstract statistical concepts, math anxiety, negative attitude, and avoidance. A survey of statistics instructors revealed that students find it more difficult to understand interrelated concepts and to apply knowledge flexibly, rather than merely performing calculations. This difficulty is attributed to the abstract nature of statistical reasoning, which requires students to generalize and connect ideas beyond surface-level understanding.^2^ Statistics courses are frequently associated with high levels of student anxiety and negative perceptions. Many students enter these courses with apprehension, believing that research and statistics are inherently difficult or irrelevant to their interests. This mindset can impede learning and reduce motivation, leading to poorer performance and avoidance of statistical content.^3^ Research indicates that graduate level students, particularly in the social sciences, often prefer qualitative over quantitative research methods. This preference is linked to a lack of confidence in statistical skills and a perception that statistics are less accessible or more challenging. Such avoidance can limit students’ research capabilities and their ability to engage with data-driven findings.^4^ These challenges are particularly significant in fields like public health, where statistical literacy is essential for interpreting data and making informed decisions. Without a strong statistical education, misinterpretation of data can lead to ineffective or misguided public health strategies. To address these challenges in statistics and a broad range of other subjects, project-based learning (PBL) has been consistently recommended over time in the education literature.^3,5–9^

PBL and traditional exams are 2 distinct methods of evaluating student performance. Each approach has its own advantages and challenges, impacting student learning, engagement, and performance. While exams typically assess knowledge recall under shorter time constraints, PBL emphasizes critical thinking and creativity through real-world application. Understanding the strengths and limitations of both methods can help educators design more effective and meaningful learning experiences across academic levels.

Project-based learning is a form of active learning that immerses students in practical, real-world challenges, deepening their comprehension and enabling them to apply concepts across various disciplines. PBL can increase student motivation and interest in general subject matter.^10,11^ A meta-analysis encompassing 66 studies over the 2 decades prior to 2023 found that PBL significantly improves students’ academic achievement, affective attitudes, and thinking skills.^12^ Another meta-analysis of 30 studies over 2 decades prior to 2017 reported a large mean effect size (*δ* = 0*.*71) for student achievement when comparing PBL to traditional instruction.^13^

Essential skills for mastering statistics and other disciplines such as critical thinking, creativity, and meta-cognition can be developed using PBL.^10^ Research also indicates that PBL increases students’ statistical literacy levels, particularly in data representation.^14,15^ Students engaged in PBL in college-level probability and statistics courses report higher satisfaction and motivation, appreciating the opportunity to apply concepts through projects, which aids in grasping statistical concepts effectively.^15^ Specifically, over 90% of these students rated their learning experience as good or excellent, appreciating the opportunity to implement projects that enhanced their grasp of statistical concepts and showed significantly improved performance in posttests and examinations. Wickramasinghe et al^16^ also found that in college-level statistics courses, PBL approaches led to increased student engagement and improved performance. A multidisciplinary, project-based introductory statistics course has been found to promote continued interest in the field, as evidenced by students’ subsequent course selections in statistics and data analysis.^17^

When integrated with programming, student outcomes can be enhanced in statistics education using PBL. Students participating in PBL reported higher confidence in applying statistical methods and interpreting data, skills crucial when using statistical software tools like R for data analysis. Lisa et al^18^ compared traditional and PBL-based introductory statistics courses and revealed that students in the PBL course reported greater confidence in statistical skills, including selecting appropriate significance tests for hypothesis testing, managing data, and writing code. An increased interest in enrolling in higher-level statistics courses was also observed among these students. Incorporating R programming into biostatistics courses equips students with essential skills for the age of artificial intelligence (AI) because of its broad use in statistics, bioinformatics, machine learning, etc. Additionally, the interdisciplinary applicability and professional readiness provided by R training make it a valuable component of modern biostatistics education to better prepare students for the evolving demands of AI and data-driven research environments.

Generally, PBL is more effective when conducted in groups rather than individually. Working on PBL projects collaboratively can lead to notable gains in computational thinking, creativity, and the overall quality of student work compared to completing projects individually.^19^ Group learning promotes mutual assistance and cooperation, mobilizing individual initiative and enhancing overall performance.^20^ Collaborative learning generally leads to better learning outcomes than individual learning, as it allows students to benefit from diverse perspectives and collective problem- solving.^21,22^ However, the success of group learning depends on factors such as group size, team formation strategies, and individual accountability. Small groups (4-5 people) have been found to be most effective for PBL, providing optimal learning outcomes.^12^ The effectiveness of group learning can be influenced by team formation strategies, such as mixed grades and complementary skills, which significantly enhance individual performance within teams.^23^ The success of group learning depends heavily on group composition and interaction. Poor group dynamics can hinder the benefits of collaborative learning.^22^ Ensuring individual accountability within group projects is crucial to prevent issues where some members do not contribute equally.^24^

Traditional written exams have been a staple in educational assessment for many reasons. They provide a consistent and standardized method for evaluating student performance, ensuring that all students are assessed under the same conditions.^25^ Exams provide a standardized way to measure student understanding, retention of course material, and assess proficiency in specific learning outcomes.^26,27^ A broad set of skills can be assessed by written exams including critical thinking, problem-solving, and the ability to articulate complex ideas clearly and coherently.^28^ They are effective in evaluating a student's depth of knowledge and understanding of the subject matter, as students must recall and apply information without external aids.^29^ Written exams give students the opportunity to articulate their reasoning and solutions in their own words, which is particularly beneficial in fields like engineering where visualizing and experimenting is crucial.^30^ This benefit also applies to the fields of statistics and data science when performing exploratory analysis or statistical inference. The need to prepare for written exams encourages students to develop good study habits and a thorough understanding of the material, as they cannot rely on external resources during the exam.^29^ Traditional exams can be more secure and easier to monitor for cheating compared to some digital formats, which can be susceptible to technical issues and unauthorized assistance.^31^

Relying solely on traditional written exams for student assessment can present several significant drawbacks. These drawbacks span various aspects of educational effectiveness, equity, and the development of essential skills. Traditional exams often emphasize memorization rather than deep understanding and critical thinking.^29,32^ Such an approach may hinder the growth of higher-order cognitive skills and reduce the overall educational value. Written exams may not effectively assess how well students can transfer and apply their knowledge to real-world situations.^33^ Written exams can be particularly challenging for nonnative English speakers, potentially disadvantaging them and affecting their performance negatively. Traditional exams can induce anxiety for students, which may affect their performance and overall learning experience. Additionally, their performance may not accurately reflect their knowledge. This disconnect between expected and actual grades has been noted in business statistics courses.^34,35^ Onwuegbuzie et al^36^ also found that anxiety negatively impacts a student's ability to conduct an appropriate statistical analysis and make statistical interpretations. This highlights the need for alternative assessment methods to ensure fair evaluation.^37^ Traditional exams may not fully gauge essential professional skills such as communication or the ability to synthesize and apply information in complex contexts. These skills require more comprehensive evaluation methods that go beyond the constraints of timed, resource-limited written exams.^38^

Studies have shown that students tend to perform better with PBL compared to traditional exams. For instance, in an engineering course, students achieved higher average marks on projects than on exams, and their learning experience improved due to the practical applications of the course content.^11^ Existing research provides valuable insights into the benefits of PBL in this context. Table 1 highlights the educational benefits of PBL compared to traditional written exams. PBL tends to improve students’ attitudes towards statistics more than traditional exams. Students appreciate the opportunity to see practical applications of their learning.^11,26^ While PBL can enhance engagement and practical understanding, traditional exams remain effective for evaluating specific learning outcomes and ensuring content coverage.^26,27^ PBL often leads to a better learning experience and higher student satisfaction. Students appreciate the practical application of knowledge and the collaborative nature of PBL, which can alleviate anxiety associated with high-stakes testing. For example, engineering students showed improved performance and a better learning experience when traditional exams were replaced with project-based assignments.^11^ Similarly, PBL in language learning reduced anxiety and improved speaking proficiency and confidence.^39,40^

**Table 1.** Educational Benefits of Project-Based Learning Compared to Written Exams.

Feature	Projects	Exams	Source
Learning depth	Encourages deep understanding and application	Often focuses on recall and surface learning	Barron et al^41^
Skill development	Builds research, collaboration, and communication skills	Tests individual memory/comprehension under time pressure	Bell et al^32^
Real-world relevance	Mimics real-world tasks; promotes transferable skills	Rarely mirrors tasks in professional settings	Wiggins et al^42^
Student engagement	Increases engagement through autonomy and creativity	Can lead to stress and disengagement	Grant et al^43^
Assessment scope	Assesses process, product, reflection, and presentation	Focuses mainly on content knowledge	Barron et al^41^
Feedback opportunities	Allows iterative feedback and revision	Typically summative with little opportunity for revision	Kolodner et al^44^
Collaboration	Encourages teamwork and peer learning; tasks can be shared	Usually completed individually	Bell et al^32^
Retention of knowledge	Promotes long-term retention due to meaningful learning	Often short-term memorization	Bell et al^32^
Creativity and innovation	Supports original thinking and creative approaches	Constrained by format and time limits	Wiggins et al^42^
Equity and accessibility	Allows multiple ways to demonstrate learning	Disadvantages such as test anxiety or different learning styles	Grant et al^43^

While PBL can alleviate anxiety, it can also provoke it depending on various factors. Research indicates that while PBL fosters deeper understanding and engagement, it can also introduce stressors related to public speaking, group dynamics, and open-ended tasks. For instance, students may experience anxiety due to the ambiguity of projects or the pressure of peer collaboration.^45^ However, several resources and strategies can help mitigate stress and anxiety among students. Grading tools such as rubrics provide clear criteria and standards for assignments so that students understand expectations, which also give them the opportunity to improve their work before final submission. Some benefits of rubrics include equitable and fair grading of student work, a reduction in the amount time spent on grading student work, effective and meaningful feedback to students, and an improvement in student learning by communicating clear expectations.^46,47^ Breaking down projects into smaller milestones allows students to manage their time better and reduces the overwhelming feeling of having a single, large deadline.^48^ Encouraging early submission of milestones can positively impact overall project performance and student behavior.^49^ Providing examples of previous student work can serve as a guide and inspiration, helping current students understand the quality and scope expected.^50^ Providing opportunities for students to receive feedback can help students to improve their projects and feel more confident about their final product. This can be achieved through instructor feedback at key stages of the project or by providing class time for students to collaborate and offer feedback to each other.^51^ Allowing students to choose their project topic can enhance their motivation by providing them with a greater sense of ownership in their work.^52,53^ Collaborative learning environments where students work together can reduce anxiety by providing social support and diverse perspectives. This interaction helps students feel less isolated and more confident in their problem-solving abilities.^54^

To the best of our knowledge, there is limited published research directly examining the impact of collaborative PBL on public health students enrolled in biostatistics courses that incorporate programming in R. However, evidence from related disciplines suggests that this approach holds significant promise for potential benefits in health- related programs where applied skills are critical. In the present study, we compared 2 cohorts of students enrolled in the same introductory biostatistics course during the Fall 2023 and Fall 2024 semesters. Both courses required programming in R at a beginner's level. The Fall 2023 cohort was evaluated using traditional written exams at both the midterm and final, whereas the Fall 2024 cohort replaced the midterm exam with a collaborative, project-based assessment while retaining the same final exam format. Our objective is to demonstrate that collaborative PBL is a compelling and effective alternative to traditional assessment methods in biostatistics education for public health students, particularly in courses emphasizing computational tools like R.

## Methods

### Design

We conducted a retrospective study comparing student performance and course evaluations of 2 cohorts of graduate students enrolled in an introductory biostatistics course with programming in R. The study duration spanned 2 academic semesters (Fall 2023 and Fall 2024) during which the course was offered to 2 independent student cohorts.

In Fall 2023, 1 instructor taught a 1-credit course in R programming while a separate instructor taught a 3-credit biostatistics course. The programming course was designed to supplement the biostatistics course. In Fall 2024, 1 instructor taught both courses because the 2 courses were combined into a single 4-credit course (3 credits for biostatistics and 1 credit for R programming). This curricular change was informed by Fall 2023 student feedback, which indicated a preference for having the material combined into a single course taught by 1 instructor. Notably, the instructor who taught the 4-credit combined course in Fall 2024 also taught the 3-credit biostatistics course in Fall 2023. The same core biostatistical topics were covered across each semester in both courses. Assessments for Fall 2023 included written midterm and final exams in addition to homework and R programming assignments. In Fall 2024, the midterm exam was replaced by a group-based data analysis project where students worked in teams of 3 to 4 using a data set of their choice from a list of recommended data sets. The shift from a written midterm to a project-based assessment was part of an effort to promote applied learning and collaborative data analysis. The project covered the same content areas as the Fall 2023 midterm including descriptive statistics and exploratory data analysis. Assessments weights were consistent across both semesters.

Because this was a retrospective study of educational cohorts without random assignment, potential confounding due to unmeasured differences between student groups could not be eliminated. However, the same instructor for biostatistics, course content, and grading rubrics were used across semesters to minimize instructional variability and reduce information bias.

### Study Population

The study population included graduate students enrolled in the Master of Public Health (MPH) degree program at the Peter O’Donnell Jr School of Public Health (OSPH) at the University of Texas Southwestern Medical Center (UTSW) in Dallas, Texas. Participants were enrolled in a required first-semester core course in introductory biostatistics with programming in R offered during the Fall 2023 and Fall 2024 semesters. OSPH is a new school, which welcomed its first cohort of students in Fall 2023. Thus, the course has only been offered twice prior to publication. The course covered topics in both descriptive and inferential statistics. The 2 cohorts were mutually exclusive, with no student repeating the course across the 2 semesters. All students enrolled in each semester who did not withdraw were included in the analysis. Topics from descriptive statistics included summary statistics (eg mean, standard deviation, etc) and exploratory analysis of data (eg tables, plots, etc). Topics from inferential statistics included confidence intervals, parametric and nonparametric significance tests (*t*-test, Wilcoxon, Mann-Whitney, chi-squared, Fisher's exact test, *F* test, Levene's test, Shapiro-Wilk test), simple linear regression, and an introduction to logistic regression.

### Data and Sample Size

This was a retrospective observational study using data from 2 past course offerings (Fall 2023 and Fall 2024). No *a priori* sample size or power calculation was conducted, as the study included all enrolled students who successfully completed the course in each semester (*n* = 33 in Fall 2023 and *n* = 32 in Fall 2024). The sample size was determined by course enrollment and not under investigator control. As such, recruitment and randomization were not applicable.

All student data were de-identified and aggregated prior to analysis. The primary objective was to evaluate the impact of assessment format (written exam vs project) on student learning and engagement between the 2 cohorts. Quantitative outcomes included midterm scores, final exam scores, overall course grades, and averaged course evaluation scores obtained from institutional records.

In addition, students completed instructor-designed surveys to provide both quantitative and qualitative data. A beginning-of-semester survey was administered to both cohorts to assess student background and expectations. Students in the Fall 2024 cohort also completed a follow-up survey designed to evaluate the perceived effectiveness of the project-based midterm. Only de-identified, aggregated survey responses were included in the analysis.

#### Midterm Assessment

Students in the Fall 2023 semester took a traditional written exam; whereas, students in the Fall 2024 completed a collaborative project-based assessment. Both assessments covered topics in descriptive statistics and focused on both computation and interpretation of results. The course objectives for both assessments were: (1) students will be able to organize data using appropriate visualization techniques; (2) students will be able to describe meaningful relationships in the data from descriptive statistical analysis; and (3) students will be able to explain the concepts of descriptive statistics.

Students in the Fall 2023 cohort were provided output from R that was needed to answer each question on the midterm exam. Additionally, there was a brief exam review session 1 week in advance and a study guide was provided 2 weeks in advance. Students were permitted to use calculators during the exam.

For students in Fall 2024, randomly assigned groups of 3 or 4 students worked on a data analysis project that spanned the first 7 weeks of the semester. They were required to use R for all data analyses. Aside from lecture and programming labs, some class time was reserved for students to work on their projects in their groups so that there were multiple opportunities for feedback. The project was broken down into 3 milestones to make it more manageable for students to ensure a positive impact on project performance. Each milestone was evaluated with a rubric so that students received further feedback on their progress as they moved toward a final product. The students were given the rubrics at the start of the semester so that they knew what was expected of them, which gave them the opportunity to assess and improve their work.

The first milestone was due in the first 2 weeks of the semester and had 2 requirements: (1) hold a first group meeting virtually or in-person and (2) select a data set related to public health for the project. The data set was required to have at least 100 observations and at least 10 variables. Students submitted their selection via email to the course teaching assistant who used a checklist rubric to assign a score. A checklist rubric lists specific criteria or elements that must be present in the student's work, with a simple yes or no evaluation. Students received full credit for the first milestone if they simply satisfied the 2 requirements. The first milestone was worth 10% of the overall project grade.

**Figure 1.**
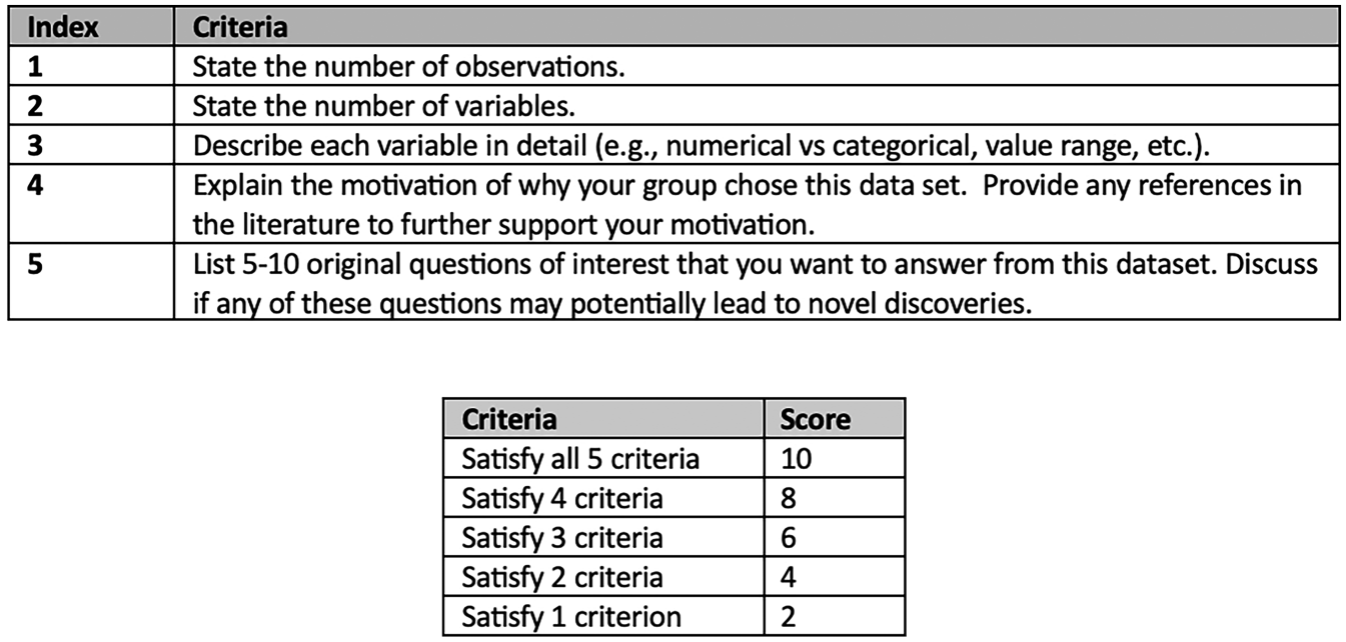
A Holistic Rubric With 5 Grading Criteria (Top). This Rubric Was Used to Score Student Proposal Papers for the Second Milestone of the Fall 2024 Midterm Project, Which Was Worth 10% of the Total Project Grade. The Score Was Based on the Number of Satisfied Criteria (Bottom).

The second milestone was due 3 weeks after the first milestone via email. The second milestone involved a written component with a 2-page limit that had 5 criteria regarding the observations, variables, their motivation behind their data set choice, and a list of research questions that they would attempt to answer using the data. The second milestone was graded by the teaching assistant using a holistic rubric (Figure 1). A holistic rubric provides a single overall score for an assessment based on an overall impression of a student's work and encourages students to focus on overall quality rather than individual criteria. The second milestone was worth 10% of the overall project grade.

**Figure 2.**
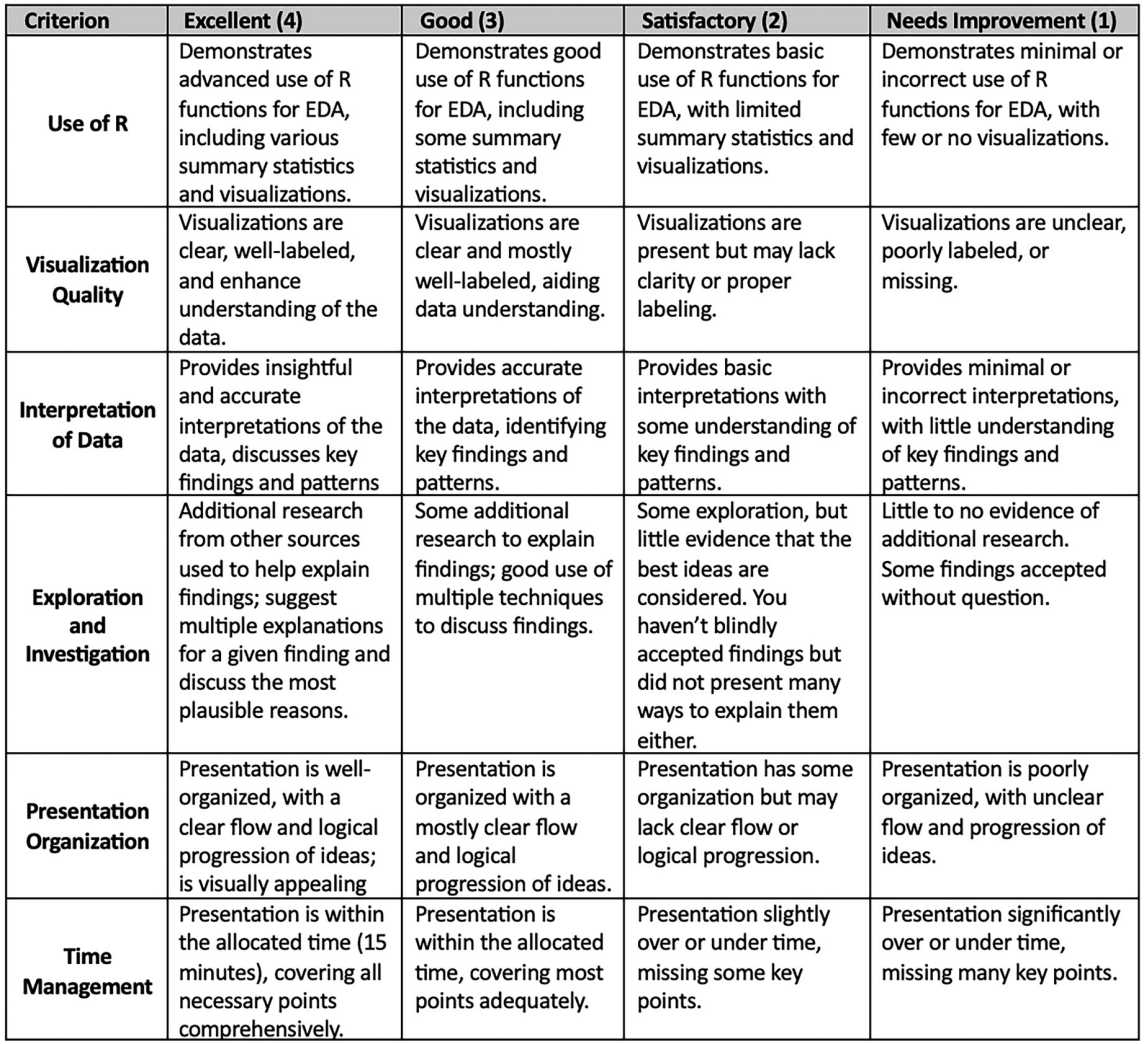
An Analytic Rubric With 6 Grading Criteria. This Rubric Was Used to Score Student Presentations for the Third Milestone of the Fall 2024 Midterm Project in a Graduate-Level Course in Introductory Biostatistics With Programming in R.

The third milestone required students to present their findings to the class using PowerPoint (or similar). Presentations had to be about 15 min in length followed by a 5-minute question and answer session. Each presentation was graded by the course instructor using an analytic rubric (Figure 2). An analytic rubric breaks down an assignment into multiple criteria, each of which is scored separately, to identify strengths and weaknesses in particular areas. This type of rubric provides detailed feedback and ensures equitable grading of all students. Lastly, students were required to email their presentation and R code files within 24 hours of the presentation. The third milestone was worth 80% of the overall project grade. Their total score for the midterm project was the sum of all milestone scores.

A document containing the full details of the Fall 2024 project including learning objectives, milestones, and grading criteria is available [Supplementary file: Project Guidelines.pdf]. Students received this document on the first day of class. Additionally, no other project-related documents were produced or distributed to students.

#### Final Exam

Students in both Fall 2023 and Fall 2024 were administered a comprehensive final exam. The exam covered topics from the entire semester, but the majority of the exam focused on statistical inference topics including confidence intervals, significance tests, hypothesis testing, and regression since these topics were covered after the midterm. Questions included, but were not limited to, asking students to interpret results in context, compute values, or determine the appropriate significance test. Analysis in R was not required for the final exam but print outs of results from R were provided for several questions. Students were permitted to use a calculator even though it was not needed to successfully complete the exam. Additionally, there was a review session about 1 week in advance and a study guide was provided 2 weeks in advance.

#### Course Grade

The same grading scheme for calculating the overall course grade was used in both Fall 2023 and Fall 2024 with respect to assessment weights. The only difference was that the midterm exam in Fall 2023 was replaced by the project in Fall 2024.

### Statistical Methods

#### Statistical Analysis

For continuous variables, summary statistics including minimum, first quartile, median, mean, third quartile, standard deviation, and variance were reported. Outliers were defined as values falling below *Q*_1_−1*.*5 × IQR or above *Q*_3_ + 1*.*5 × IQR, where *Q*_1_ denotes the first quartile or 25th percentile, *Q*_3_ denotes the third quartile or 75th percentile, and IQR denotes the interquartile range (IQR = *Q*_3_−*Q*_1_). The Mann-Whitney *U* test was used to compare Fall 2023 and Fall 2024 distributions due to the nonnormality of the data. The 95% confidence interval (CI) and *P*-value of the *U* test for the difference in group medians were reported. Levene's test was used to test for homogeneity of group variances; *P*-value and variance ratio were reported. The variance ratio compares the variance of Fall 2023 (numerator term) to Fall 2024 (denominator term). For categorical variables, proportions are reported. Fisher's exact test was used to compare qualitative variables for Fall 2023 and Fall 2024. Additionally, 95% confidence intervals for the difference of group proportions were reported along with the *P*-values from Fisher's exact test. All significance tests were 2-sided with a type I error rate of 5%. There were no missing data. All statistical analyses were conducted in R and RStudio version 4.3.3.

#### Sensitivity Analysis

Because all students in each group of the project-based cohort received the same midterm score, we conducted a sensitivity analysis to account for the lack of independence among group members so that group scores from Fall 2024 are not overrepresented compared to Fall 2023 exam scores. Specifically, we selected 1 representative midterm score per group (*n* = 10 groups) and compared these to the Fall 2023 midterm exam scores (*n* = 33 students). Given the small sample size and nonnormality in the data, we first used the nonparametric Mann-Whitney *U* test to compare the 2 groups. To further explore the group difference while addressing potential limitations in statistical power of nonparametric methods due to small sample size, we also conducted a Bayesian quantile regression analysis using the brms package in R. The outcome variable was midterm score, and the predictor was cohort (exam-based vs project-based). We modeled the posterior distribution of the group median difference using the asymmetric Laplace distribution, which is appropriate for estimating differences in medians in Bayesian analysis. Weakly informative priors were used for all model parameters *via* software default. Convergence was assessed *via* R-hat statistics, which are also known as Gelman-Rubin statistics. From the posterior distribution, we computed the posterior probability that the median score in the project-based cohort exceeded that of the exam-based cohort and obtained a 95% credible interval for the difference in medians. This Bayesian approach provided a complementary estimate of group median differences, particularly under conditions of small sample size and nonnormality.

### EQUATOR Guidelines

This study is reported in accordance with the STROBE guidelines (Strengthening the Reporting of Observational Studies in Epidemiology) [Supplementary file: STROBE checklist.pdf].

## Results

### Student Characteristics

Students took a survey at the beginning of the semester so that the instructor could qualitatively assess and compare the educational background of each group. There were no statistically significant differences between Fall 2023 and Fall 2024 students. While there were more females than males, the proportions of females and males between semesters were not significantly different. Specifically, 82% of Fall 2023 students and 75% of Fall 2024 students were female (*P* = *.*56, 95% CI: [−0.16, 0.30]). The majority of students who attended another academic institution within 5 years of starting the MPH program was 76% of the students in Fall 2023 and 74% of the students in Fall 2024 (*P* = *.*78, 95% CI: [−0.210, 0.255]). The proportion of students who had previously taken statistics elsewhere was 83% of the students in Fall 2023 and 97% of the students in Fall 2024 (*P* = *.*11, 95% CI: [−0.32, 0.03]). For those who had previously taken statistics elsewhere, 68% from Fall 2023 and 53% from Fall 2024 had taken the course at least 5 years prior (*P* = *.*29, 95% CI: [−0.138, 0.428]). The proportion of students who had never used R was 94% for the students in Fall 2023 and 81% of the students in Fall 2024 (*P* = *.*20, 95% CI: [−0.056, 0.326]). In summary, the 2 cohorts were very similar in many respects.

### Academic Performance

**Figure 3.**
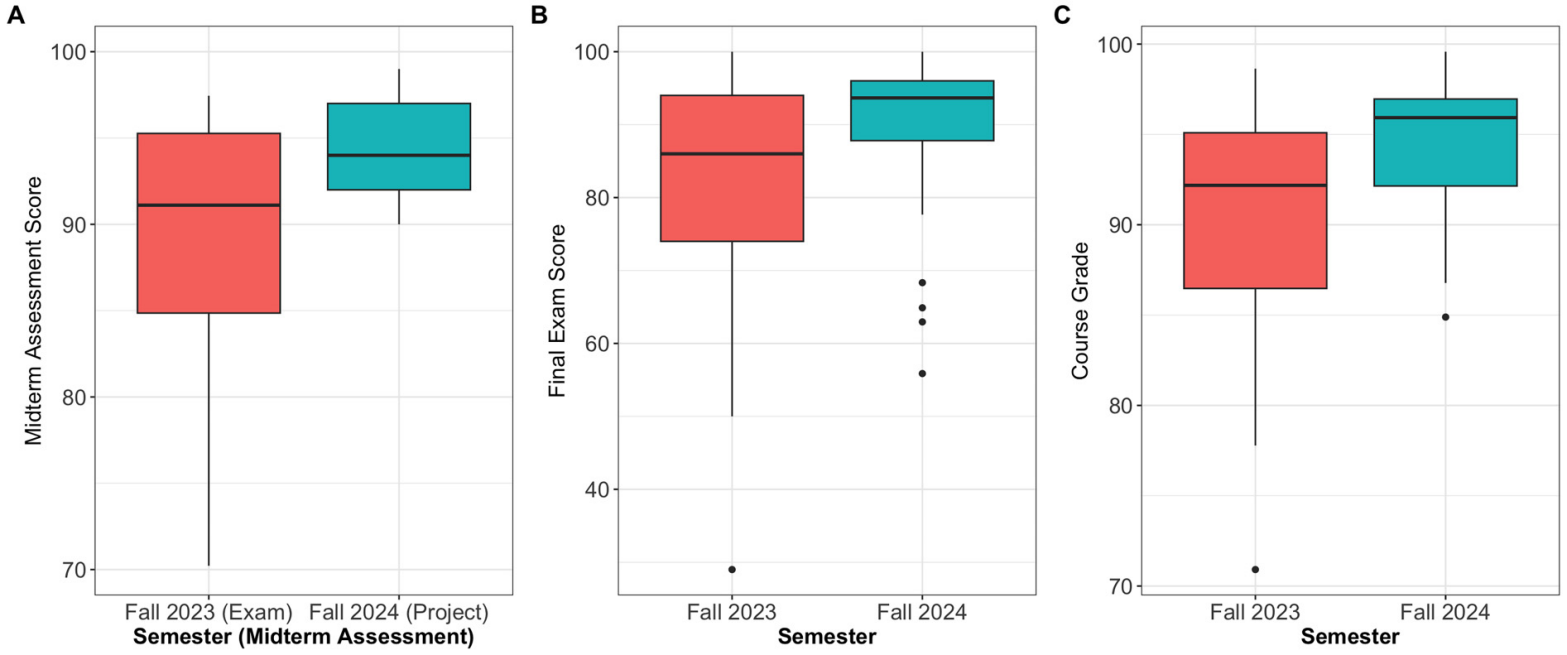
Side-by-Side Boxplots. Plot A: Midterm Assessment Scores Grouped by Semester and Assessment (*P* = .01 for Individual Student Scores (*n* = 32); *P* = .07 for Individual Group Scores (*n* = 10)). The Plots Are Identical in Both Cases; Plot B: Final Exam Scores Grouped by Semester (*P* = .03); Plot C: Course Grades Grouped by Semester (*P* = .002). Each Dot Represents an Outlier.

To evaluate the effect of assessment structure on student performance, we compared midterm assessment scores (exam vs project), final exam scores (exam vs exam), and overall course grades between the 2 cohorts: Fall 2023, which employed a traditional written midterm exam, and Fall 2024, which implemented a project-based assessment in place of the midterm. Descriptive statistics are presented in Table 2 and side-by-side box-and-whisker plots of the assessment score distributions by assessment are presented in Figure 3. The Fall 2024 cohort demonstrated statistically significantly higher midterm scores compared to the Fall 2023 cohort. The median score for students completing the project-based midterm was 94*.*0, as opposed to 91*.*1 for those completing the traditional midterm exam (*P* = *.*01, 95% CI: [−6.7, −0.8]) as illustrated in Figure 3A. Additionally, the variability in student performance was substantially lower in Fall 2024 (variance = 9*.*0) compared to Fall 2023 (variance = 57*.*0), a difference that was statistically significant (*P* *<* *.*001, variance ratio = 6*.*7). These findings suggest that the project-based assessment may offer a more equitable and consistent measure of student learning outcomes. It should be noted that all groups received full credit for the first 2 milestones, which accounted for 20% of the total midterm score. Thus, the variability in project-based midterm scores was entirely due to performance on the final milestone (presentation and code submission), which accounted for 80% of the total midterm score.

**Table 2.** Summary Statistics for Midterm, Final Exam, and Course Grades by Cohort.

	*n*	Min	*Q*_1_	Median	Mean	*Q*_3_	Max	SD	Variance
Midterm									
Fall 2023 (exam)	33	70.2	84.9	91.1	89.2	95.3	97.5	7.6	57.0
Fall 2024 (project): by individual	32	90.0	92.0	94.0	94.1	97.0	99.0	3.0	9.0
Fall 2024 (project): by group	10	90.0	92.0	94.0	94.1	97.0	99.0	3.2	10.1
Final exam									
Fall 2023	33	29.0	74.0	86.0	81.2	94.0	100.0	18.0	329.9
Fall 2024	32	55.9	87.8	93.7	89.2	96.0	100.0	11.4	131.0
Course grade									
Fall 2023	33	70.9	86.5	92.2	90.2	95.1	98.6	6.9	47.9
Fall 2024	32	84.9	92.1	95.9	94.8	97.0	99.6	3.6	13.0

Abbreviations/Notations: Sample Size *(n)*, Minimum Score (Min), First Quartile *(Q1)*, Third Quartile *(Q3)*, Maximum Score (Max), and Standard Deviation (SD).

To account for the group-based grading structure in the project-based cohort, we conducted a sensitivity analysis comparing only 1 midterm score per group (*n* = 10) to individual midterm exam scores from the exam-based cohort (*n* = 33). The Mann- Whitney *U* test yielded a *P*-value of *.*07 for this comparison, suggesting a trend toward higher scores in the project-based cohort, though not reaching statistical significance. Given the potential for low statistical power of nonparametric methods when sample size is small, we followed up with a Bayesian quantile regression model targeting the median group difference, using the asymmetric Laplace distribution. All R-hat statistics were equal to 1 indicating good model convergence. The posterior probability that the project-based cohort had higher median midterm scores than the exam-based cohort was 93%. The Bayesian model estimated the project-based midterm median to be 1*.*67 points higher than the exam-based group. However, the 95% credible interval for the group median difference (−0.72, 4.16) included zero, reflecting some uncertainty about the magnitude and direction of the effect.

Although the final exam format remained constant between cohorts, students in the Fall 2024 section achieved significantly higher scores than the Fall 2023 section. The median score was 86*.*0 in Fall 2023 compared to 93*.*7 in Fall 2024 (*P* = *.*03, 95% CI: [−10.0, −0.5]) as illustrated in Figure 3B. While there was no statistically significant difference in variance (*P* = *.*08, variance ratio = 2*.*5), the median score shifted meaningfully, suggesting that the benefits of project-based learning may extend to summative assessments that follow a project-based assessment.

Final course grades likewise favored the Fall 2024 project-based cohort. The median course grade was 92*.*2 in Fall 2023 compared to 95*.*9 in Fall 2024 (*P* = *.*002, 95% CI: [−6.7, −0.8]). Moreover, the distribution of grades was significantly more consistent in Fall 2024 (variance = 13*.*0) compared to Fall 2023 (variance = 47*.*9) (*P* = *.*009, variance ratio = 3*.*7). These results reinforce the positive impact of the project-based approach on overall course performance and grade equity compared to courses with only a traditional approach.

### Fall 2024 Postproject Survey

To further evaluate the effectiveness of the project-based assessment as well as solicit both positive and constructive feedback, students in the Fall 2024 cohort were invited to complete a postproject survey. This survey was administered following the midterm project presentations and sought to capture student perceptions of their learning experience, confidence with course content, group collaboration experience, and the relevance of the project to real-world data analysis.

#### Checklist-Related Questions

**Figure 4.**
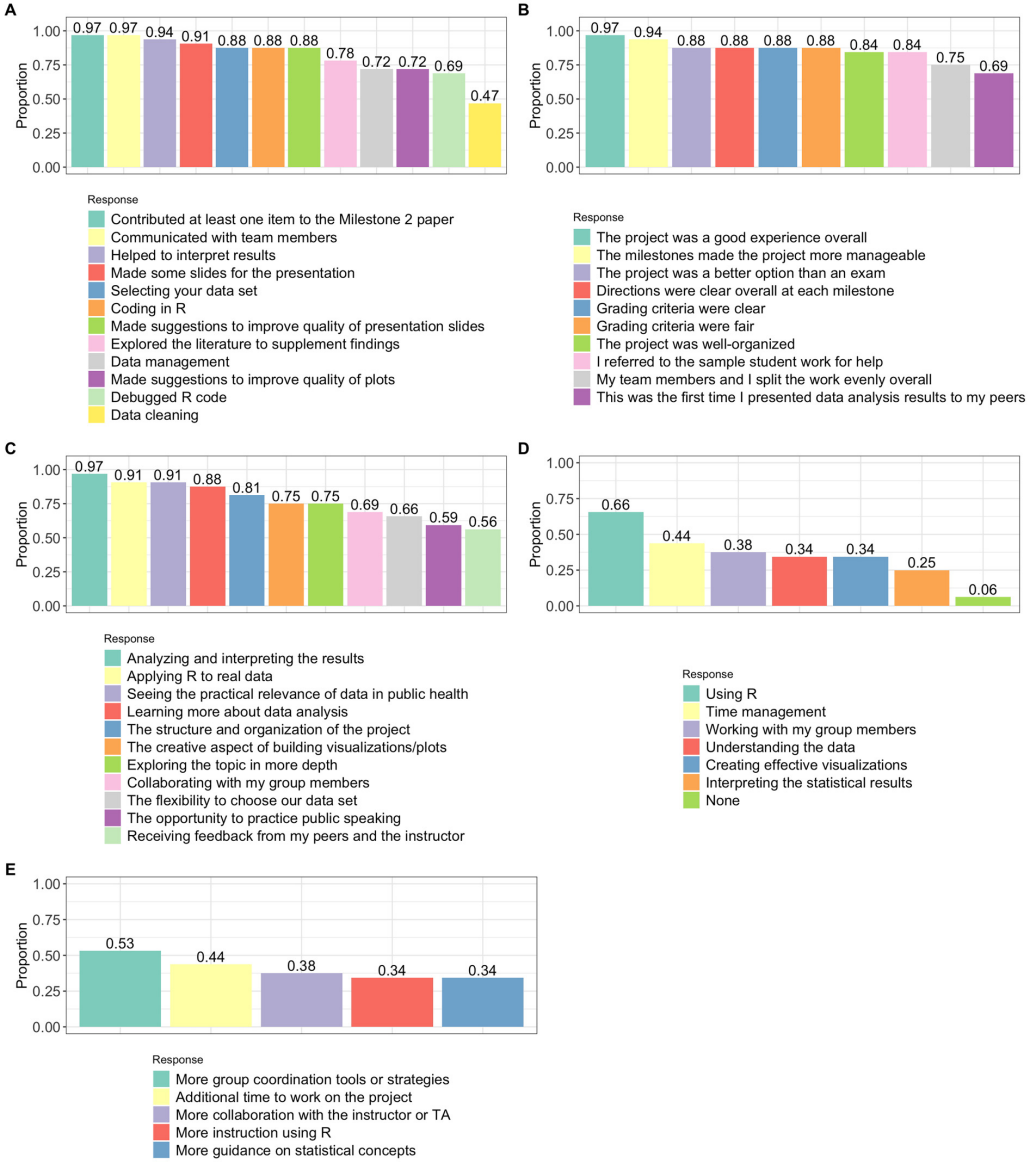
Responses to the Checklist-Related Questions. The Height of Each Bar Gives the Proportion of Students Who Selected the Response Corresponding to That Category. Students Could Select Multiple Answers to Each of These Questions. Subplot Letters Correspond to Question Letters in the Main Text. The Responses Are Ordered From Left to Right Along the Horizontal Axis Starting With the Category at the Top of the Legend.

Students were asked to respond to several questions about the project with the option to select more than 1 response per question. Figure 4 displays the proportion of students who selected each response. Subfigure letters correspond to the question letters below:

- “Describe your overall contributions to the project. Select all that apply.” [Note: Students had the option to type in other contributions that were not listed.]
- “Which of the following statements do you agree with about the midterm project? Select all that apply.”
- “What did you like about the project? Select all that apply.”
- “What was challenging about the project? Select all that apply.”
- “What support or resources would have helped you more during this project? Select all that apply.”

Responses to checklist-style questions indicated strong student engagement in the project. In question A, the majority of students reported active participation in group meetings, data analysis, R programming, and preparation of the final presentation. Notably, more than 3 quarters of the class contributed to the analytical components of the project, with students citing individual efforts such as code debugging, documentation, and organizing communication platforms (eg, Microsoft Teams). Even though less than half contributed to data cleaning, most of the data sets were analysis-ready when the students selected them. Additional but optional responses manually submitted by students included:

- “Compiled the code for submission, added comments explaining the code, edited the code for consistency and clarity.”
- “Once all the data was completed, I also double checked it to make sure it ran through completely, with no issues. Afterwards, I ran any changes by the group before making edits where they were needed.”
- “Create Microsoft teams group.”

When asked to reflect on the project's value in question B, most students agreed that the experience was well-designed, practical, and relevant to their future work in public health. Approximately two-thirds reported that this was their first experience presenting the results of a real data analysis, underscoring the opportunity for authentic professional skill development. However, students also reported common challenges, including limited prior experience with R (81% had never used the software), time management difficulties, and inconsistent effort from team members. These responses suggest the importance of structuring support mechanisms to promote equity and accountability in group-based PBL settings.

Responses to question C reflected a range of opinions. While more than half of the students indicated that they appreciated all aspects of the project, the highest levels of endorsement were for its practical and applied nature. In contrast, fewer students expressed enjoyment of components such as group collaboration, public speaking, and receiving feedback during presentations. This may likely reflect underlying anxiety related to these activities. Question D revealed that the most commonly cited challenge was programming in R, which is consistent with expectations given that 81% of the students had no prior experience with the software. The second most frequently reported difficulty was time management, a common hurdle for first-semester graduate students adapting to new academic and workload demands. Question E responses further underscored difficulties related to team dynamics and time constraints. While 97% of students had previously completed a statistics course, approximately one-third still felt additional instructional time would have been beneficial. This may reflect lingering math anxiety, gaps in prior preparation, or challenges related to balancing workloads in a fast-paced graduate environment.

#### Rate-Related Questions

**Figure 5.**
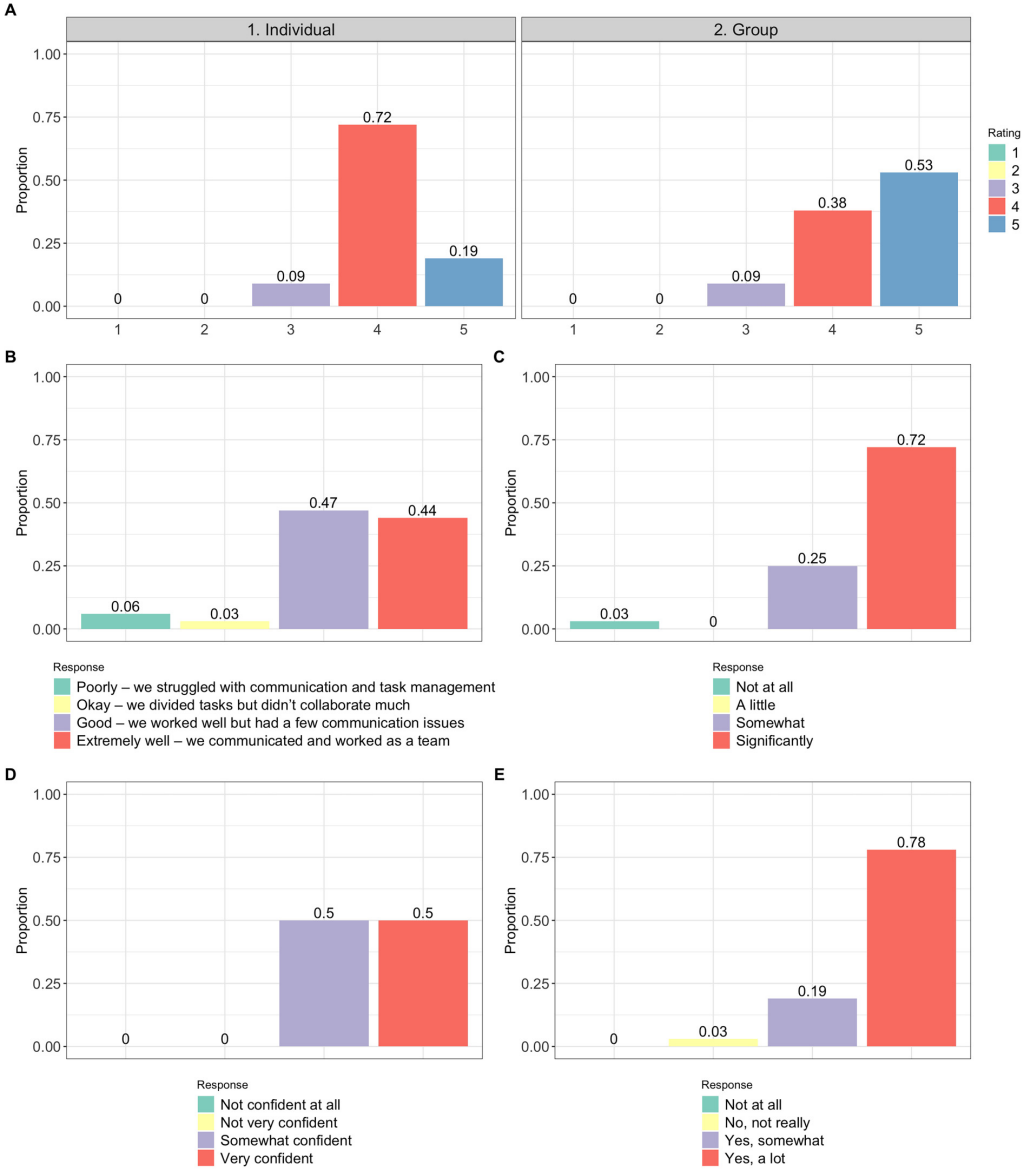
Responses to the Rate-Related Questions. The Height of Each Bar Gives the Proportion of Students Who Selected the Rating Corresponding to That Bar. Students Could Only Select 1 Rating per Question. Subplot Letters Correspond to Question Letters in the Main Text. The Responses Are Ordered From Left to Right Along the Horizontal Axis Starting With the Category at the Top of the Legend.

Students were asked to rate several items about the project. Figure 5 displays the proportion of students who selected each rating, which was based on either a 4 or 5 point scale depending on the question. Students were only able to select 1 rating per question. Subfigure letters correspond to the question letters below:

1. “Rate your overall performance as an individual during the presentation (1 is the lowest which suggests improvement is needed and 5 is the highest which suggests great performance).” See the “Individual” panel of Figure 5A.
2. “Rate your group's overall performance during the presentation (1 is the lowest which suggests improvement is needed and 5 is the highest which suggests great performance).” See the “Group” panel of Figure 5A for results.
3. “How well did your group collaborate?”
4. “How much did this project enhance your understanding of data analysis in R?”
5. “How confident did you feel presenting your findings?”
6. “Do you feel the project helped you better understand real-world applications of data analysis?”

Students rated their own and their group's performance highly, with the mode score being a 4 for individual performance and a mode score of 5 for group performance (on a 5-point scale). This result suggests that students generally viewed their group's overall effort as slightly stronger than their own individual contributions. These ratings reflect perceptions of performance, not necessarily preferences regarding collaborative versus individual work. Other survey responses, such as appreciation for peer support and overall experience of the group project, suggest that a majority of students found value in the group-based structure of the project. Nearly all respondents reported that the project improved their understanding of data analysis in R, boosted their confidence in presenting results, and helped them recognize the real-world relevance of biostatistics. These findings align with the educational goals of project-based learning and offer additional evidence of its positive impact on developing both technical and interpersonal skills.

#### Optional Question

Students were also asked but not required to answer the following prompt: “Please make any suggestions that can help improve the project for future cohorts.” Some positive feedback included:

- “I feel like this really helped me with my coding because I was forced to do it all myself. I learn more when given a blank slate and then a okay go do it and this project really pushed me to do that. Since I had to figure out the coding pretty much by myself.”
- “I was glad that my group used a real dataset. Working with a real data set makes it more realistic training for the real world and makes it more interesting because there are real implications from the data.”

The positive feedback both here and from elsewhere in the survey in general validates what the literature supports for PBL. Students emphasized the value of autonomy in learning and the challenge of problem-solving from a “blank slate,” which aligns with research showing that PBL promotes deeper learning and critical thinking through student-driven inquiry. Additionally, the use of real-world data helped students see the relevance of their work beyond the classroom, a key feature of authentic learning experiences that enhance motivation and engagement. These responses suggest that when students are given meaningful, context-rich tasks, they not only build technical skills but also develop confidence in applying those skills independently. Next, some constructive feedback included:

- “I’d break up the presentations in two days.”
- “It should be a requirement that each group member should contribute at least one graph for the presentation.”
- “I am not opposed to teamwork. I understand collaboration in public health, but I didn’t expect to encounter similar issues here as I did in undergraduate studies, where not everyone took the work as seriously as others or leading to situations where one person ends up doing all the project.”
- “Maybe allowing students to choose their own teams might also help in ensuring better collaboration.”
- “As this is a group project, I suggest adding one more requirement: each group should schedule at least three meetings independently before the final presentation. This will promote better communication among group members and help everyone stay on track throughout the project.”

The constructive feedback highlights several important considerations for implementing collaborative PBL effectively. While students recognized the value of collaboration, they also pointed to common challenges in group work such as uneven participation, communication breakdowns, and time management. These challenges can hinder the overall experience. Suggestions like requiring individual contributions, setting expectations for group meetings, and allowing team self-selection underscore the need for clearer structure and accountability within group dynamics, which was also evident in aforementioned survey question responses. Additionally, logistical concerns, such as presentation scheduling, suggest that pacing and workload distribution play a role in how students perceive and engage with the project. Overall, the feedback affirms that while PBL has many strengths, its success depends on careful and intentional planning that addresses the interpersonal and organizational complexities of collaborative work. These comments highlight areas for future iteration of the course design, particularly with respect to managing group dynamics and balancing structure with flexibility.

### Course Evaluations

Average course evaluation scores increased from 4*.*1 in Fall 2023 to 4*.*7 in Fall 2024, representing a 15% improvement in overall student satisfaction. Given that other course components such as lecture content, final exam format, homework assignments, and programming instruction remained largely unchanged, this improvement is likely attributable to the introduction of the project-based midterm. Taken together, the enhanced academic performance, encouraging postproject survey responses, and higher course evaluation scores suggest that project-based learning offers a more effective approach for teaching, assessing, and preparing students in data analysis within an introductory biostatistics course using R.

## Discussion

This study examined the impact of replacing a traditional written midterm exam with a collaborative, project-based assessment in an introductory graduate-level biostatistics course with programming in R for graduate-level public health students. Key findings suggest that students in the project-based cohort outperformed their exam-based peers in midterm and final exam scores and demonstrated more consistent overall course performance. Survey responses further indicated increased confidence in coding and data analysis, higher satisfaction with the course experience, and appreciation for the opportunity to apply biostatistical concepts to real-world public health data. In conclusion, this study supports the integration of well-structured project-based assessments as a pedagogical strategy to enhance student learning and engagement in introductory biostatistics courses.

While our primary analysis showed a statistically significant increase in midterm scores for the project-based cohort, a sensitivity analysis using 1 score per group did not meet the conventional threshold for significance. Given the small number of observations in the project cohort (*n* = 10 groups), this result may reflect limited statistical power rather than a lack of effect. The high posterior probability from the Bayesian analysis suggests moderate evidence in favor of improved performance in the project-based group, though with some residual uncertainty. Together, these findings indicate that the apparent benefits of project-based assessment on midterm performance may have been partly influenced by group grading structure and sample size limitations. Nevertheless, consistent improvements observed in final exam performance and course evaluations, both individually assessed and unaffected by group structure, support the broader conclusion that project-based learning may have positively influenced student engagement and achievement.

The observed improvements in academic performance and student engagement support the growing body of literature suggesting that PBL approaches foster deeper learning and skill development, particularly in applied disciplines like public health. The structured use of rubrics, milestone-based feedback, and data analysis tasks likely contributed to these outcomes by enhancing transparency, motivation, and accountability.

However, several limitations should be noted. The retrospective, nonrandomized design introduces potential for confounding. *A priori* sample size or power analysis was not feasible for this retrospective study, as we used existing data from 2 full course cohorts. While this approach ensured complete data coverage, the relatively small sample size, especially in the sensitivity analysis using 1 score per group, may have limited our ability to detect small but meaningful differences between cohorts. Although student demographic and academic backgrounds were largely similar between cohorts, unmeasured factors such as cohort-specific motivation, external stressors, or differing peer dynamics may have influenced outcomes. The instructor for the biostatistics course was the same in both semesters, which helps control for variability in teaching style, but combining the R programming and biostatistics content into 1 integrated course in Fall 2024 may have introduced subtle curricular changes not fully accounted for. In addition, the use of different midterm assessment formats (exam vs project) complicates direct comparisons of scores, as the methods may evaluate different skill sets. Although the project and exam covered the same statistical topics, the nature of assessment likely emphasized different cognitive processes.

Potential sources of bias include potential differences in group collaboration quality and unequal distribution of effort within teams, as acknowledged by students in the postproject survey. Social loafing, scheduling conflicts, and varying levels of statistical or programming proficiency may have influenced individual and group performance. While rubrics were designed to support equitable grading, challenges related to peer dynamics remain an inherent limitation in collaborative learning environments. The higher and more consistent midterm scores in the project-based cohort may be partly explained by the assessment structure. For example, all groups received full credit for the first 2 milestones, which accounted for 20% of the total project score. As a result, only the presentation (Milestone 3, worth 80%) contributed to score variation. While this structure was intentional and pedagogically grounded in principles of scaffolding and formative feedback to encourage student engagement, it may have introduced a ceiling effect that partially contributed to the reduced variability and higher overall midterm scores in the project-based cohort. Nevertheless, higher final exam and course evaluation scores were observed in the project-based cohort. Further research in diverse settings and with randomized designs will help refine our understanding of when and how PBL is most effective.

Despite these limitations, the findings provide cautious yet compelling evidence that project-based assessments can serve as effective alternatives to traditional exams in biostatistics education. The improvements observed in final exam performance suggest that PBL may also have a positive spillover effect on subsequent summative assessments, potentially through enhanced comprehension and retention of material. Both project-based assessments and traditional exams have their merits in introductory statistics courses. Project-based assessments can enhance engagement, practical application, and skill development, while traditional exams provide a standardized measure of student proficiency. Written exams still serve a purpose, especially for quickly assessing a wide breadth of knowledge or ensuring standardization across large groups. But project-based learning shines when the goal is to build real- world competencies and deeper learning. The choice between these methods should consider the specific goals of the course, student needs, and the resources available for implementation.

In terms of generalizability, our results are most applicable to similar educational settings: graduate-level public health programs where students have limited prior experience with R or statistical analysis. The structured and supportive design of the PBL assignment, including clearly defined milestones and feedback mechanisms, may be essential to reproducing positive outcomes. Institutions considering similar curricular changes should account for the resources required to support collaborative learning, including instructor and teaching assistant availability, rubric design, and facilitation of group work.

## Conclusion

This study found that incorporating a collaborative project-based assessment in place of the traditional midterm significantly improved student performance, engagement, consistency in grades, and overall course satisfaction. Survey results also indicated increased confidence with statistical programming in R, improved understanding of data analysis, and stronger collaboration among peers. Student feedback emphasized the value of real data and hands-on learning, while also pointing to areas for improvement such as group dynamics and time management. These findings support the integration of structured, well-supported project-based learning as a valuable pedagogical strategy in biostatistics education, particularly in areas such as public health training programs where applied skills are critical.
